# Antimicrobial resistance among *Escherichia coli* isolates from dogs presented with urinary tract infections at a veterinary teaching hospital in South Africa

**DOI:** 10.1186/s12917-018-1552-7

**Published:** 2018-07-31

**Authors:** Daniel Nenene Qekwana, Lufuno Phophi, Vinny Naidoo, James Wabwire Oguttu, Agricola Odoi

**Affiliations:** 1 Section Veterinary Public Health, Department of Paraclinical Sciences, Faculty of Veterinary Science, University of Pretoria, Pretoria, South Africa; 2 Biomedical Research Centre (UPBRC), Faculty of Veterinary Science, University of Pretoria, Pretoria, South Africa; 3 Department of Agriculture and Animal Health, College of Agriculture and Environmental Sciences, University of South Africa, Johannesburg, South Africa; 4 Department of Biomedical and Diagnostic Sciences, College of Veterinary Medicine, University of Tennessee, Knoxville, USA

**Keywords:** Antimicrobial resistance, E.coli, Urinary tract infections, Dog, Canine, Multidrug resistance, Extensive drug resistance, Pan-drug resistance

## Abstract

**Background:**

This study investigated the burden and predictors of canine *E. coli* urinary tract infections (UTI) and antimicrobial resistance among dogs presented at a veterinary teaching hospital in South Africa, 2007–2012.

**Methods:**

The Cochran-Armitage trend test was used to investigate temporal trends while logistic regression models were used to investigate predictors (age, sex, breed, year) of *E. coli* infections and antimicrobial resistance (AMR).

**Results:**

A total of 22.3% (168/755) of the urinary specimens tested positive for *E. coli*. A significant (*p* = 0.0004) decreasing temporal trend in the percentage of *E. coli* positive isolates was observed over the study period. There were high levels of AMR to penicillin-G (99%), clindamycin (100%), tylosine (95%), cephalothin (84%) but relatively low levels of resistance to enrofloxacin (16%), orbifloxacin (21%). Almost all (98%, 164/167) the isolates exhibited multidrug resistance (MDR), while only 11% (19/167) and 2% (4/167) exhibited extensive drug resistance (XDR) and pan-drug resistance (PDR), respectively.

**Conclusions:**

Although, the risk of *E. coli* UTI declined during the study period, the risk of AMR increased. The high levels of AMR and MDR as well as the presence of XDR and PDR is concerning as these have the potential of affecting prognosis of UTI treatments.

## Background

Although recent studies show that *Enterococcus* spp. and *Pseudomonas spp.* are increasingly becoming prominent in urinary tract infections (UTI) in dogs, *Escherichia coli* remains the most common cause of UTI in dogs [1–4]. These infections are caused by uropathogenic *E. coli* (UPEC), which differ from intestinal *E. coli* strains as they contain extra virulence genes, allowing a successful transition from the intestinal tract to the urinary tract [5]. The perianal and genital areas are the principal reservoirs of the *E.coli* organism known to cause UTI [5]. Animals with compromised immune systems are at higher risks of UTI than those that are not immune-compromised. In these animals, the organisms are able to multiply and persist in a portion of the urinary tract resulting in clinical disease [6, 7]. Urinary tract infections can be divided into upper and lower tract infections. The former affects the kidneys and ureters while the latter affects the bladder, urethra and vagina [2, 3, 8–10]. Clinical signs of *E. coli* UTI in dogs may include acute cystitis, pyelonephritis and urosepsis. These three clinical signs are distinct indicators of UTI syndromes [1].

Antimicrobial agents such amoxycillin-clavulanic acid, tetracyclines, trimethoprim-potentiated sulphonamides and cephalexin are reported to be effective against *E. coli* UTI [11–13]. However, there are concerns of increased antimicrobial resistance among *E. coli* isolates to fluoroquinolones in dogs with UTI [1]. Unfortunately, there is limited information on the burden and predictors of antimicrobial resistance (AMR), multidrug resistance (MDR) and extensive drug resistance (XDR) among companion animals in South Africa. This is despite evidence of transfer of resistance between animals and their owners. Therefore, the objective of this study was to investigate the burden of *E. coli* infections and antimicrobial drug resistance among dogs presented with UTI at a veterinary teaching hospital in South Africa.

## Methods

### Data source and management

This study used retrospective data from the bacteriology laboratory of a veterinary teaching hospital in South Africa. Client owned dogs from Gauteng Province with suspected UTI whose specimens were tested at the laboratory between January 2007 and December 2012 were included in the study. Since this study used retrospective laboratory records, it did not directly involve animals and thus posed no risk to client animals. The data were assessed for duplicates and missing information. Only complete records were selected for inclusion in this study. The following variables were extracted from the records: age (in months), sex, breed and the date of specimen submission. The breed classification used in the study was adapted from the American Kennel Club (AKC) and included the following categories: working, sporting, herding, hound, toy, terrier, nonsporting and mixed breeds [14].

### *Escherichia coli* identification and antimicrobial susceptibility testing

*Escherichia coli* were isolated and identified using standard bacteriological methods and suspected *E. coli* colonies were subjected to different biochemical tests as described by Quinn et al. [15]. The *E. coli* reference strain (ATCC 25922) was used for quality control.

*E. coli* isolates were subjected to antimicrobial susceptibility testing against a panel of 15 drugs using the disc diffusion method (Kirby-Bauer method). The panel included the following antibiotics: amikacin (30 μg), doxycycline (30 μg), enrofloxacin (5 μg), gentamicin (10 μg), ampicillin (10 μg), penicillin G (10 μg), trimethoprim-sulphamethoxazole (co-trimoxazole) (25 μg), chloramphenicol (30 μg), cephalothin (30 μg), kanamycin (30 μg), clindamycin (2 μg), lincospectin (lincomycin hydrochloride and spectinomycin sulfate) (100 μg), orbifloxacin (5 μg), Synulox (amoxicillin/clavulanic acid) (20/10 μg) and tylosin (15 μg) (Oxoid Ltd., Cambridge, UK). To determine the susceptibility profiles of the isolates, the bacteriology laboratory that processed the specimens followed the Clinical and Laboratory Standards Institute (CLSI) procedures for isolation, testing and classification (2013, Clinical Institute Laboratory Standards 2007, Clinical Institute Laboratory Standards 2011, Clinical Institute Laboratory Standards 2010, Clinical Institute Laboratory Standards 2012, Clinical Institute Laboratory Standards 2008, Clinical Institute Laboratory Standards 2009). Based on the laboratory assessments, *E. coli* isolates were classified as Susceptible, Intermediate or Resistant. Isolates that exhibited intermediate resistance were re-classified as resistant. Multidrug resistance (MDR) was defined as resistance to at least one agent in more than three antimicrobial categories [16]. Extensive drug resistance (XDR), on the other hand, was defined as resistance to all but two of the tested antimicrobial agents in each category while pan-drug (PDR) resistance was defined as resistance to all antimicrobial categories tested [16].

### Data analysis

#### Descriptive analysis

Crude and factor-specific proportions of *E. coli* UTI and AMR as well as their 95% confidence intervals were computed. The factors assessed were age, sex, breed and year. Associations between UTI and AMR and each of the above factors were assessed using the Chi-square or Fishers Exact tests as appropriate. The temporal trends in the proportions of *E. coli* UTI and AMR between 2007 and 2012 was assessed using Cochran–Armitage trend tests. Significance was set at α = 0.05 for all statistical tests.

#### Predictors of infection

The predictors of *E. coli* UTI were assessed using logistic regression models. A simple binary logistic regression was first fit to assess the association between infection status (yes/no) and covariates age, sex, breed and year. Predictors with a *p*-value less than 0.20 were considered for inclusion in the multivariable logistic regression model. A manual backwards elimination approach was then used to build a multivariable logistic regression model containing variables that had potential univariable associations (*p* < 0.2) with the outcome. At this stage significance was set at α = 0.05. To assess for confounding, the changes in parameter estimates of the predictors in the model with and without the suspected confounding variable were compared. Changes of 20% in the estimates were considered indicative of significant confounding and hence the suspected confounding variables were retained in the final model. Adjusted odds ratios and 95% confidence intervals were calculated for all predictors retained in the final model. Statistical significance was assessed using Wald Chi-Squared Test at α = 0.05. Hosmer-Lemeshow test was used to assess goodness-of-fit of the final model.

## Results

### Descriptive analysis

The median age of dogs tested was 72 months (Interquartile range: 32–116). More males (58%) than females (42%) were tested (Table 1). The working breeds constituted the highest proportion (24%) of dogs tested followed by the hound (14%), herding (13%) and sporting (13%) breeds. The highest proportion (23%) of specimens was tested in 2010, followed by 2007 and 2009 each with 20% specimens tested (Table 1).

**Table 1:** Profile of all specimens tested for *Escherichia coli* urinary tract infections at the bacteriology laboratory of a veterinary teaching hospital in South Africa, 2007–2012

Variable	Number of Specimens	Percentage of Specimens
Sex (*n* = 676)		
Female	393	58.1
Male	283	41.9
Breed (*n* = 677)		
Working	162	23.9
Hound	97	14.3
Herding	89	13.2
Sporting	87	12.9
Terrier	85	12.6
Toy	80	11.8
Nonsporting	45	6.7
Crossbreed	32	4.7
Year (*n* = 683)		
2010	156	22.8
2007	137	20.1
2009	137	20.1
2008	110	16.1
2011	97	14.2
2012	46	6.7

### Risks of *Escherichia coli* UTI

Twenty two percent (22%; *n* = 168/755) of the urinary specimens tested positive for *E. coli.* Based on simple association assessments, there was no significant association between risk of *E. coli* UTI and breed (*p* = 0.283). On the other hand, there was a significant association between risk of *E. coli* UTI and both year (*p* < 0.001) and sex (*p* = 0.054) (Table 2).

**Table 2:** Distribution of the proportion *Escherichia coli* Urinary Tract Infections by sex, breed and time among dogs admitted to a veterinary teaching hospital, 2007–2012

Variable	Number Tested	Number Positive	Percentage Positive	*P*-values
Sex				
Female	393	80	20.36	0.054
Male	283	50	17.67	
Breed				
Working	162	43	26.54	0.283
Hound	97	16	16.49	
Herding	89	17	19.1	
Sporting	87	17	19.54	
Terrier	85	17	20	
Toy	80	10	12.5	
Nonsporting	45	7	15.56	
Crossbreed	32	6	18.75	
Year				
2007	137	39	28.47	<0.001
2008	110	27	24.55	
2009	137	19	13.87	
2010	156	29	18.59	
2011	97	19	19.59	
2012	46	0	0	

### Antimicrobial resistance

The majority of *E. coli* isolates were resistant to penicillin-G (99.4%), clindamycin (100%), tylosine (95.0%), cephalothin (84%), amoxycillin-ampicillin (70%), doxycycline (68%) and lincospectin (63%). However, low levels of resistance were observed against enrofloxacin (16%), orbifloxacin (21%), trimethoprim-sulphamethoxazole (25%) and chloramphenicol (25%) (Table 3).

**Table 3:** Antimicrobial resistance patterns of *Escherichia coli* from urine specimens of clinical cases of dogs admitted to a veterinary teaching hospital, 2007–2012

Antimicrobial	Number Tested	Number Resistant	Percent Resistant
Penicillin G	164	163	99.4
Clindamycin	160	160	100
Tylosine	161	153	95.0
Cephalothin	166	139	83.7
Amoxycillin	164	115	70.1
Doxycycline	166	112	67.5
Lincospectin	164	104	63.4
Synulox	163	95	58.3
Kanamycin	163	91	55.8
Amikacin	166	61	36.8
Gentamicin	166	47	28.3
Trimethoprim-Sulphamethoxazole	166	41	24.7
Orbifloxacin	162	34	21.0
Chloramphenicol	118	29	24.6
Enrofloxacin	167	27	16.2

Resistance to Lincosamides (100%), Lincospectin (100%), Macrolide (95%), Cephalosporin (84%), Penicillin (70%), Tetracycline (68%) was very high. On the contrary, much lower resistance levels were observed against Amphenicol (25%), Aminoglycoside (22%) and Fluoroquinolone (13%) (Table 4). With regard to multiple resistance, almost all *E. coli* isolates that were AMR exhibited MDR (98%, 164/167), while 11% (19/167) were XDR and only 2% (4/167) were PDR.

**Table 4:** Antimicrobial resistance of *Escherichia coli* isolates from urine specimens of canine clinical cases admitted to the veterinary teaching hospital, 2007–2012

Antibiotic Class	Number Tested	Number Resistant	Percent Resistant
Lincosamides	160	160	100
Lincospectin	104	104	100
Macrolides	161	153	95.0
Cephalosporins	166	139	83.7
Penicillins	167	117	70.1
Tetracyclines	166	112	67.5
Synulox	163	95	58.3
Potentiated sulpha	166	41	24.7
Amphenicols	118	29	24.6
Aminoglycoside	167	36	21.6
Fluoroquinolone	167	21	12.6

#### Predictors of *Escherichia coli* infection and antimicrobial resistance

Based on the multivariable logistic model, age (*p* = 0.465), sex (*p* = 0.318) and breed (*p* = 0.300) all showed no evidence of significant association with odds of *E. coli* UTI. However, there was a significant association between the odds of *E. coli* UTI and time (years) with the odds of infection significantly (*p* < 0.001) decreasing (OR = 0.78, 95% CI: 0.68–0.89) during the study period.

None of the variables assessed: age (*p* = 0.972), sex (*p* = 0.282), breed (*p* = 0.309) and year (*p* = 0.394) had a significant association with the odds of multi-drug resistance among *E. coli* isolates.

## Discussion

In this study, we investigated the burden and predictors of *E. coli* UTI and their antimicrobial resistance patterns from dogs presented at a veterinary teaching hospital in South Africa. The results of this study will support management and treatment of UTIs in dogs presented at the veterinary hospital.

In this study, we observed a lower proportion of *E. coli* positive samples (22%) than the 27% reported by Stiffler et al. [17], 56% reported by Seguin et al. [13], 44% reported by Johnson et al. [2] in the USA and 62% reported by Gibson et al. [1] in Australia. The differences in the results may be due to differences in study designs. The studies by Stiffler et al. [17] and Seguin et al. [13] were longitudinal studies investigating risk before hospitalization and after surgery, while our study investigated *E. coli* UTI among hospitalized dogs in a veterinary teaching hospital. It is also possible that the presence of underlying disorders in the study by Stiffler et al. [17] and Seguin et al. [13], which our study did not investigate, could explain the differences in the proportions of *E. coli* positive samples. Dogs with underlying medical conditions such as diabetes mellitus, hyper-adrenocorticism and pre-existing urinary tract diseases have a weaker immune system making them prone to *E. coli* infections [13].

Thompson et al. [4] reported an increase in the prevalence of UTI in dogs over the period of their study. However, in our study, we observed a decrease in the proportion of *E. coli* positive samples. This could be due to improved health and welfare of the dogs visiting the hospital.

The results of this study show that age, breed and sex were not significantly associated with the odds of *E. coli* UTI among dogs presented at the hospital in South Africa. This is consistent with findings by Stiffler et al. [17] who reported no significant association between *E. coli* UTI and age, breed or weight of the dog. However, Stiffler et al. [17] reported that female dogs were 3 times more likely to contract *E. coli* related UTI compared to male dogs. By contrast, Johnson et al. [2] observed that the risk of *E. coli* UTI was higher in males compared to female dogs. These findings suggest that there may be sex predisposition for *E. coli* related UTI although our study did not identify such a relationship. Sex related risk of *E. coli* UTI has been shown to be related to the differences in the anatomic structure between male and female dogs. This makes manually expressing of female bladder easier for urine sample collection than intermittent catheterization needed in male dogs [17]. Moreover, indwelling urinary catheters during diuresis or administration of corticosteroid are also major risk factors of UTI in dogs. These may be due to conformational changes, altered normal flora, or decreased immune response [7, 17, 18]. Contrary to the findings of our study, Stiffler et al. [17] reported that dogs ≤3 years were more likely to present with UTI compared to dogs > 3 years.

While resistance to penicillin-G, clindamycin, tylosin, cephalothin, amoxycillin, ampicillin, doxycycline and lincospectin were common in the majority of the *E. coli* isolates in this study, a study in Sweden reported low levels of resistance to ampicillin (17%), and tetracycline (7%) among *E. coli* UTI [12]. The reason for the higher resistance levels observed in this study is unclear and requires further investigation. However, low levels of resistance to fluoroquinolones, sulpha trimethoprim and chloramphenicol observed in our study are similar to the findings of other studies [11, 13, 19].

Although our findings are contrary to those of Wedley et al. [20] who observed a low proportion (18%) of *E. coli* that were MDR-among dogs with urinary tract infections, the high proportion of MDR (98%) in our study is not unusual. For example, Gibson et al. [1] and Wagner et al. [21] also observed high proportions of MDR among E.coli isolates from dogs with UTIs. This observation has serious implications for clinical outcomes during treatment given that multiple drug resistance has a negative effect on the prognosis of *E. coli* UTI in veterinary medicine [22–24]. Of even greater concern among the findings of the present study is the presence of XDR (11%) and PDR (2%) *E. coli* isolates. The proportion of XDR isolates observed in this study is higher than the 2% reported by Thungrat et al. [25] in the USA. Furthermore, the ability of *E. coli* isolates to transfer resistance genes among themselves and other species of bacteria [26], makes the levels of XDR and PDR observed in this study of great veterinary public health concern.

In the present study, no previous history of antimicrobial usage among the dogs tested was available. Moreover, some cases might have been treated empirically prior to culture and susceptibility testing. In addition, isolates that exhibited intermediate resistance in this study were re-classified as resistant. Therefore, it is possible that this could have slightly biased the findings towards higher estimates of resistance levels among the *E. coli* isolates observed in this study. Nonetheless, the results of this study support previous studies that showed that *E. coli* is a common cause of UTI in dogs and contributes to the understanding of antimicrobial resistance patterns among *E. coli* UTI in the dog population presented at the veterinary teaching hospital in South Africa.

## Conclusions

This study shows that the proportion of cases of *E. coli* UTI among dogs presented at the veterinary teaching hospital declined over the study period. However, high levels of *E. coli* isolates exhibiting MDR, XDR and PDR are of clinical and a veterinary public health concern. Therefore, urgent action needs to be taken to tackle the development of antimicrobial drug resistant *E. coli* infections in dogs. This may require development of antimicrobial stewardship programme at the teaching hospital and in the country.

## Abbreviations

AKC: American Kennel Club
AMR: Antimicrobial resistance
CLSI: Clinical and Laboratory Standards Institute
E.coli: *Escherichia coli*
PDR: Pan-drug resistance
UPEC: Uropathogenic *E. coli*
UTI: Urinary tract infections
XDR: Extensive drug resistance
